# Efficacy and safety of oral propranolol and topical timolol in the treatment of infantile hemangioma: a meta-analysis and systematic review

**DOI:** 10.3389/fphar.2024.1515901

**Published:** 2024-12-02

**Authors:** Xiaoye Huang, Wenyue Si, Zhili Zou, Binyao Li, Yide Mu, Wei Zhong, Kaiying Yang

**Affiliations:** ^1^ Department of Pediatric Surgery, Guangzhou Women and Children’s Medical Center, National Children’s Medical Center for South Central Region, Guangzhou Medical University, Guangzhou, China; ^2^ School of Pediatrics, Guangzhou Medical University, Guangzhou, China; ^3^ Department of Science Research and Education Management, Guangzhou Women and Children’s Medical Center, National Children’s Medical Center for South Central Region, Guangzhou Medical University, Guangzhou, China; ^4^ The First School of Clinical Medicine, Guangzhou Medical University, Guangzhou, China

**Keywords:** infantile hemangioma, propranolol, timolol, efficacy and safety, meta-analysis

## Abstract

**Background:**

Propranolol, a nonselective β-blocker, is the first-line treatment for infantile hemangioma (IH). Topical timolol has recently been proposed as a novel IH treatment with fewer adverse effects. This study was conducted to compare the efficacy and safety of oral propranolol and topical timolol for treating IH.

**Methods:**

Studies were included after searching PubMed, Embase, Web of Science, and the Cochrane Library via the keywords of “propranolol”, “timolol”, “infantile hemangioma” and their synonyms. A meta-analysis with pooled odds ratios was performed using the fixed-effect model.

**Results:**

Seven articles with 2071 patients were included in this meta-analysis. Compared with topical timolol, oral propranolol had a greater response rate (OR = 2.12, *P* < 0.001), but it was also associated with a greater risk of adverse events (OR = 2.31, *P* < 0.001). For superficial IH, timolol demonstrated similar efficacy to propranolol (OR = 1.28, *P* = 0.34) but with fewer adverse events (OR = 2.30, *P* = 0.001). Additionally, compared with topical timolol, propranolol at a dosage of 2 mg/kg/d had a better response rate (OR = 2.62, *P* < 0.001), whereas the 1.0∼1.5 mg/kg/d propranolol group showed no significant difference (OR = 1.34, *P* = 0.38).

**Conclusion:**

Oral propranolol presents superior therapeutic efficacy in the treatment of IH compared to topical timolol. However, topical timolol can serve as an alternative to oral propranolol for treating superficial IH, providing similar efficacy with fewer adverse effects. Additionally, propranolol at a dosage of 2 mg/kg/d offers greater efficacy with a comparable safety profile, whereas the 1.0∼1.5 mg/kg/d propranolol dosage shows no significant difference in efficacy compared to timolol but is associated with more adverse events.

**Systematic Review Registration:**

https://www.crd.york.ac.uk/prospero/display_record.php?ID=CRD42024603724, identifier CRD42024603724.

## 1 Introduction

Infantile hemangioma (IH) is the most common benign vascular tumor in children, with a prevalence of approximately 5%–10%, and predominantly affects females (Holm et al., 2024). It is mainly classified as superficial, deep, and combined lesions on the basis of the extent of skin involvement. Additionally, it can also be divided into focal, multifocal, and segmental patterns according to the number of anatomical sites involved (Rodriguez Bandera et al., 2021). IH commonly occurs in the head and neck region, with most lesions being isolated focal tumors (Mitra et al., 2024). Risk factors for IH include prematurity, low birth weight, female sex, white race, placental anomalies, and family history (Leaute-Labreze et al., 2017).

IH displays a distinctive growth pattern with a rapid proliferative phase within the first year after birth, followed by spontaneous regression that lasts for several years (Hasbani and Hamie, 2022). Without treatment, nearly 70% of the regressed IH will lead to permanent residual skin changes, such as telangiectasias, fibrofatty tissue, and atrophic skin (Leaute-Labreze et al., 2017). Although the pathogenesis of IH remains unclear, both vasculogenesis and angiogenesis, especially angiogenesis, play vital roles in the development of IH (Ji et al., 2014). As the major pathogenetic mechanism of IH, pathological angiogenesis is driven primarily by degradation of the basement membrane, followed by the proliferation, migration, and aggregation of activated hemangioma-derived endothelial cells (HemECs) to form neovasculature (Xiang et al., 2024). Overexpression of human vascular endothelial growth factor (VEGF) has been reported to be essential for the promotion and maintenance of blood vessel growth (Olsson et al., 2006). Additionally, hypoxia promotes the release of growth factors such as VEGF, fibroblast growth factor (FGF), and platelet-derived growth factor (PDGF) to stimulate angiogenesis (Mitra et al., 2024).

Propranolol, a nonselective β-blocker, can target β-1 and β-2 adrenergic receptors and suppress the expression of VEGF and its receptor, thus inhibiting lesion proliferation and inducing IH involution (Satterfield and Chambers, 2019). Currently, propranolol has replaced corticosteroids as the first-line treatment for IH (Krowchuk et al., 2019). As another nonselective β-blocker, timolol is reported to have equivalent efficacy to propranolol as a topical therapy, especially for small and superficial IH (Ovadia et al., 2015; Puttgen et al., 2016). Timolol is associated with mild and infrequent adverse events, while propranolol often leads to more frequent issues, such as sleep disturbances, cold extremities, and gastrointestinal symptoms (Lin et al., 2020; Leaute-Labreze et al., 2016). While most studies report higher response rates for propranolol, Chelleri et al. found that patients treated with timolol had the lowest rate of residual lesions, suggesting its potential advantage in specific cases (Chelleri et al., 2020).

Therefore, with the aim of identifying more appropriate treatment options for the management of IH, this meta-analysis was conducted to compare the efficacy and safety of oral propranolol and topical timolol by incorporating the literature more comprehensively. Additionally, we performed subgroup analyses, including comparisons of the safety and efficacy of these treatments for superficial and non-superficial IH, as well as evaluations of the differences in treatment outcomes between varying propranolol dosages (1.0∼1.5 mg/kg/d and 2 mg/kg/d) and topical timolol.

## 2 Materials and methods

### 2.1 Study design

This meta-analysis was conducted according to the PRISMA 2020 (Preferred Reporting Items for Systematic Reviews and Meta-Analyses) statement and the Cochrane Handbook for Systematic Reviews and Meta-analyses (Page et al., 2021).

### 2.2 Search strategy

The search was performed in PubMed, Embase, Web of Science, and the Cochrane Library with the keywords “propranolol”, “timolol”, “infantile hemangioma” and their synonyms. The search was conducted on August 6, 2024, which was completed within 1 day. Two researchers (XY Huang and WY Si) independently conducted searches in the database, removed duplicates via Endnote X9, and screened the studies based on the inclusion and exclusion criteria. Any disagreements encountered were resolved through discussion with the other researcher.

### 2.3 Study selection

Studies that met the following criteria were included: (1) published in English and open access; (2) patients with IH; (3) direct comparisons of the efficacy and safety of oral propranolol and topical timolol in IH; and (4) valid data, including definitive results of efficacy and safety and any adverse events that occurred during the treatment. Studies were excluded if (1) the type of study was not original research, including review, letter, conference abstract, note, or editorial; (2) the study was a duplication; or (3) patient characteristics were not reported.

### 2.4 Data extraction

Two researchers (XY Huang and WY Si) independently extracted the data and summarized them in an Excel sheet, including title, author, publication year, country, number of samples, patient characteristics (sex ratio, mean age, tumor site, clinical classification, follow-up time, and mean treatment duration), and intervention factors (dosage, efficacy and number of adverse events). A variety of indicators can be used to assess efficacy, including the Hemangioma Activity Score (HAS) (Janmohamed et al., 2011), the Visual Analog Scale (VAS) (Puttgen et al., 2016), and the Achauer’s 4-point scale (Achauer et al., 1997). In our research, the VAS score ranging from 0 to 100 or a tumor reduction in size of less than 50% were considered ineffective treatments. Adverse events included both systemic adverse reactions and all drug-related side effects mentioned. Patients who responded ineffectively to topical timolol and were subsequently treated with oral propranolol were enrolled in both timolol and propranolol treatment.

### 2.5 Quality assessment

Two researchers (ZL Zou and W Zhong) independently evaluated the quality of all included articles and resolved all discrepancies by consensus. We evaluated the quality of the randomized controlled trials (RCTs) via the Cochrane risk of bias tool and the non-RCTs via the ROBINS-I tool (Babic et al., 2024; Sterne et al., 2016). The Newcastle–Ottawa Quality Assessment Scale (NOS) was used to evaluate the observational studies, with a score of ≥7 indicating high-quality studies, a score of 4–6 indicating moderate-quality studies, and a score of 0–3 indicating low-quality studies (Wells et al., 2021). Disagreements were resolved through discussion with the corresponding author.

### 2.6 Statistical analysis

Heterogeneity was assessed by *I* ^*2*^ statistics and the Cochrane *Q* statistic. *I* ^*2*^ statistics values above 50% and *P* < 0.10 indicate significant heterogeneity (Higgins et al., 2003). When heterogeneity is low (*I* ^*2*^ < 50%), it is assumed that the true effect size is consistent across all studies, and a fixed-effects model was used. However, when heterogeneity is high (*I* ^*2*^ > 50%), the effect sizes may vary between studies due to differences in study characteristics, such as populations, interventions, and methodologies. In such cases, a random-effects model was used to provide a more conservative and reliable estimate of the overall effect size (Borenstein et al., 2010). Forest plots were drawn with Review Manager 5.4, and StataMP 18 was used to evaluate publication bias and conduct sensitivity analyses. The odds ratio (OR) and 95% confidence interval (CI) were calculated for the fixed-effects models. Publication bias was estimated by funnel plots, and symmetrical scatter on the funnel plot revealed no publication bias (Lin, 2019). Sensitivity analyses were performed by omitting one study at a time.

## 3 Results

### 3.1 Summary of study selection

**FIGURE 1:**
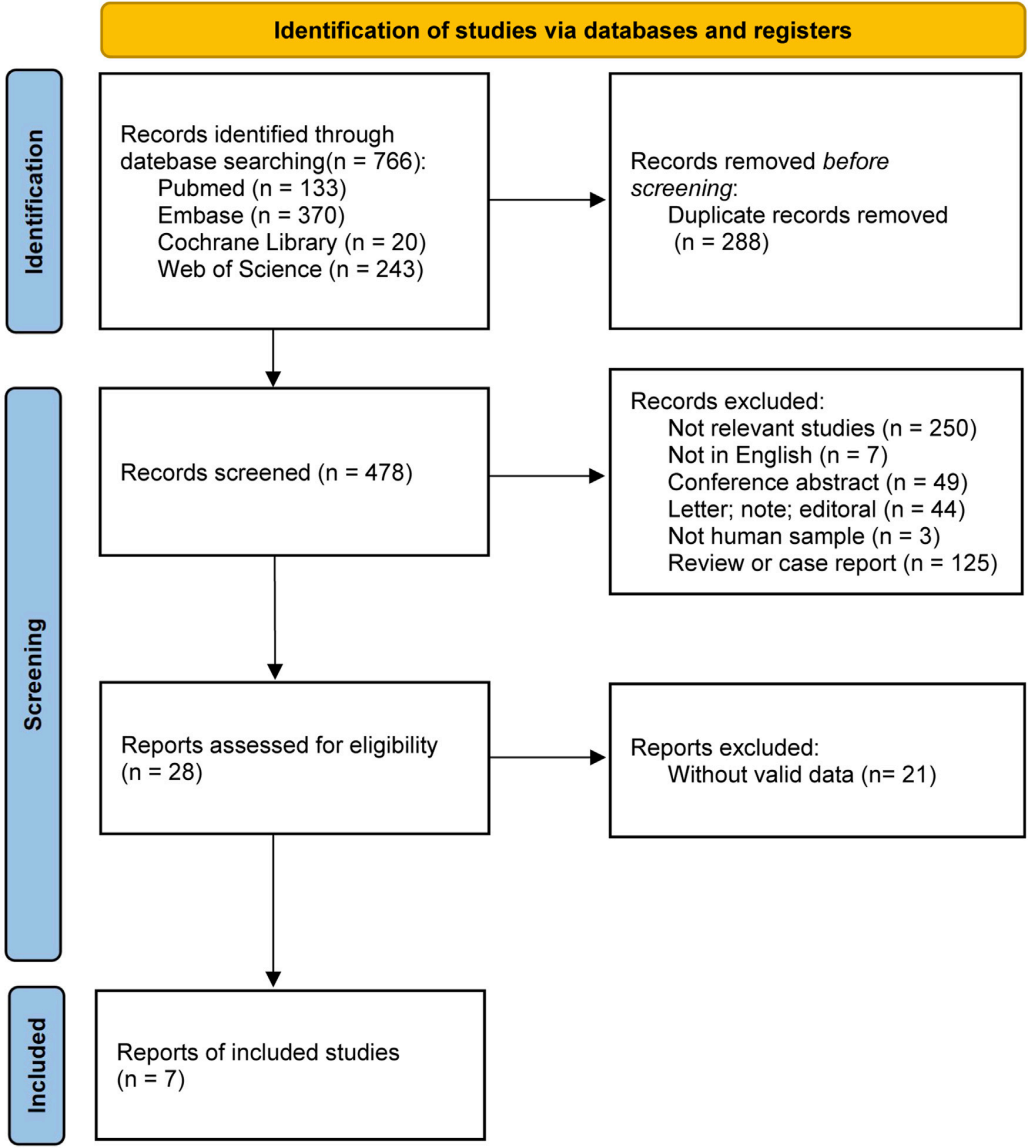
PRISMA diagram.

A total of 766 articles were retrieved from four databases according to the search strategy, including 133 from PubMed, 370 from Embase, 20 from the Cochrane Library, and 243 from the Web of Science. After removing duplicate records and screening the studies according to the inclusion and exclusion criteria, 7 articles (1 RCT, 2 non-RCTs, and 4 observational studies) were included in the meta-analysis (Figure 1).

### 3.2 Study characteristics

Overall, 2,071 patients were enrolled in the study, including 1,366 treated with oral propranolol alone, 674 treated with topical timolol alone, and 31 patients treated with oral propranolol due to ineffective treatment with timolol (Table 1). The majority of the included studies were conducted in China (5/7, 66.7%) (Gong et al., 2015; Zhang et al., 2016; Yuan and Wang, 2024; Han et al., 2024; Wu et al., 2018), with the others in the United Kingdom (Sinha and Lloyd, 2020) and Romania (Tarca, 2020). The patients included were predominantly female, with a median age of 5.1 months and a male-to-female ratio of 1:2.67. The mean treatment duration was 6.9 months, with a mean follow-up time of 15.1 months. The locations of the tumors are mainly on the head and neck, with predominantly superficial IH. The dosages of oral propranolol used include 2 mg/kg/d (Zhang et al., 2016; Wu et al., 2018; Sinha and Lloyd, 2020; Tarca, 2020) and 1.0∼1.5 mg/kg/d (Gong et al., 2015; Yuan and Wang, 2024; Han et al., 2024), and the treatment regimens for topical timolol include 0.5% solution of timolol maleate (Gong et al., 2015; Zhang et al., 2016; Yuan and Wang, 2024; Han et al., 2024; Sinha and Lloyd, 2020; Tarca, 2020) and 0.5% timolol maleate hydrogels (Wu et al., 2018).

**TABLE 1:** Characteristics of the included studies.

Studies	Country	Sample	Gender (Female: Male)	Median age	Follow-up time	Location	Clinical classification	Treatment duration
Han, 2024	China	60	1.86:1	4.8 months	6.6 months	Mainly in the head and neck	Superficial	6.4 months
Yuan, 2024	China	307	1.13:1	1.62 months	6 months	Head and face, Limbs and trunk	Superficial	6 months
Sinha, 2020	England	35	28:10	9 months	36 months	Mainly in the head and neck	Unknown	9 months
Tarca, 2020	Romania	66	2.5:1	5.7 months	21 months	Head (face or scalp), chest or abdomen, limbs, perineal, chin and multiple hemangiomas	Unknown	7 months
Wu, 2018	China	724	2.79:1	5.8 months	6.4 months	Head and neck, extremities, trunk	Superficial	6.7 months
Zhang, 2016	China	853	5.1:1	3.57 months	22 months	Face and neck	Superficial, subcutaneous, mixed	7 months
Gong, 2015	China	26	24:15	2–9 months	3–12 months	Eyelids, lips, nose, ears, parotid and cheek	Superficial	6 months

### 3.3 Study quality

The quality of the included studies was evaluated as described in the Supplementary Tables S1–S3. Selection bias, performance bias, detection bias, attrition bias, reporting bias, and other biases of the RCT (Gong et al., 2015) according to the Cochrane assessment tool are listed in Supplementary Table S1, showing a low risk of bias. For non-RCTs, the methodological quality according to the ROBINS-I tool is presented in Supplementary Table S2. Both non-RCTs (Han et al., 2024; Wu et al., 2018) had low risk levels. Concerning the quality of the other observational studies (Zhang et al., 2016; Yuan and Wang, 2024; Sinha and Lloyd, 2020; Tarca, 2020), all four studies were of high quality, with Newcastle–Ottawa Scale (NOS) scores ≥7 (Supplementary Table S3). In brief, all the included studies presented a low risk of bias and good overall methodological quality.

### 3.4 Efficacy and safety outcomes

**FIGURE 2:**
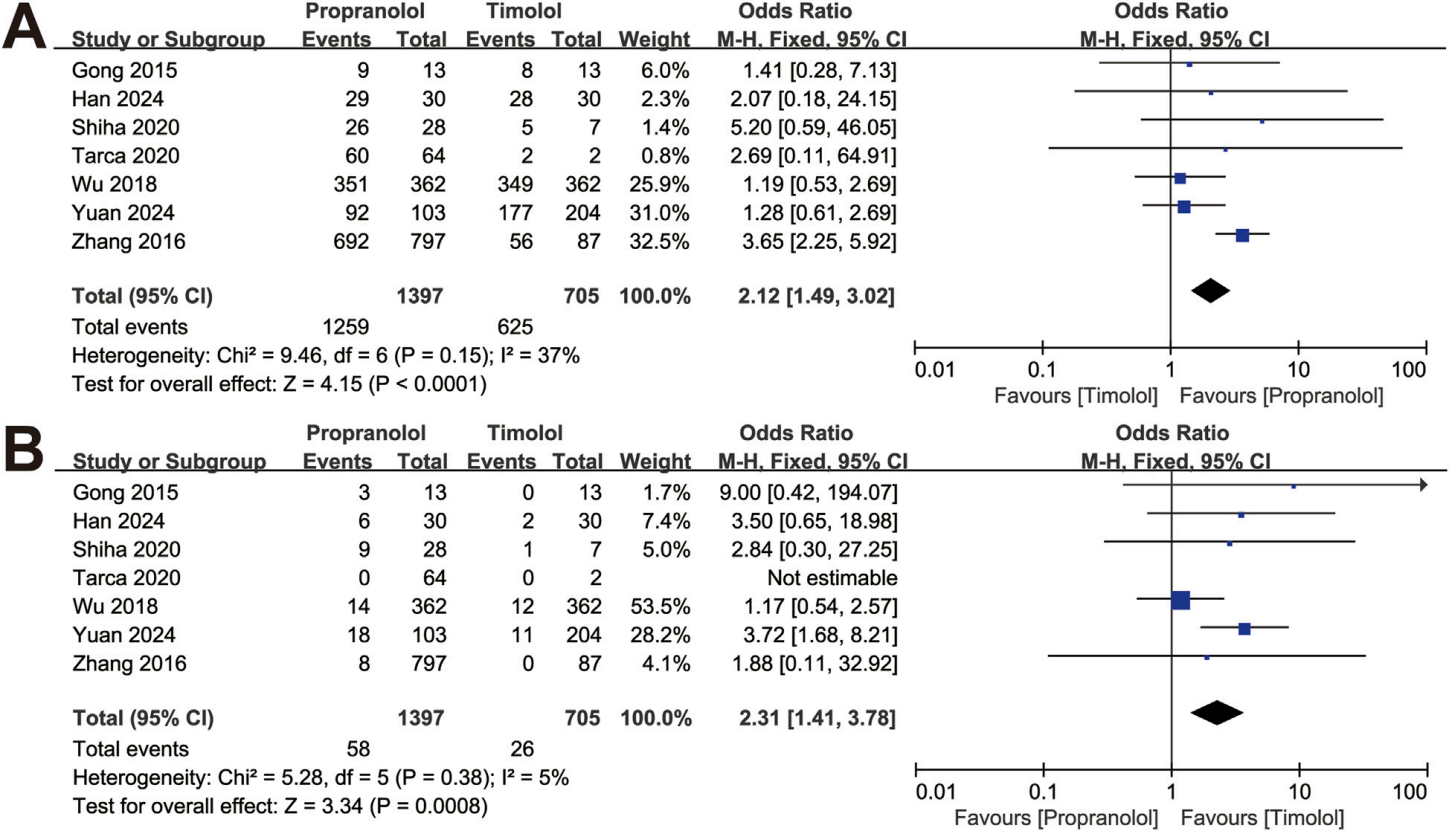
Forest plot of **(A)** efficacy and **(B)** adverse events.

In total, 1,397 patients treated with oral propranolol and 705 patients treated with topical timolol were analyzed (Figure 2). Among the included studies, 2 used the VAS score to measure treatment efficacy (Han et al., 2024; Wu et al., 2018), and 3 utilized Achauer’s 4-point scale (Gong et al., 2015; Zhang et al., 2016; Yuan and Wang, 2024). Among the 1,397 patients treated with propranolol, 1,259 were categorized as therapeutically effective, whereas 138 were categorized as having a poor response. Similarly, of the 705 patients treated with timolol, 625 patients were categorized as therapeutically effective, and 80 patients were categorized as having a poor response.

As presented in Figure 2A, the response rate of IH patients in the propranolol-treated group was greater than that in the timolol-treated group (OR = 2.12, 95% CI: 1.49–3.02; *P* < 0.001), with no significant heterogeneity (*P* for Cochrane’s Q test = 0.15, *I* ^*2*^ = 37%). The safety between the two treatment groups was significantly different (OR = 2.31, 95% CI: 1.41–3.78; *P* < 0.001; Figure 2B).

### 3.5 Subgroup analysis

**FIGURE 3:**
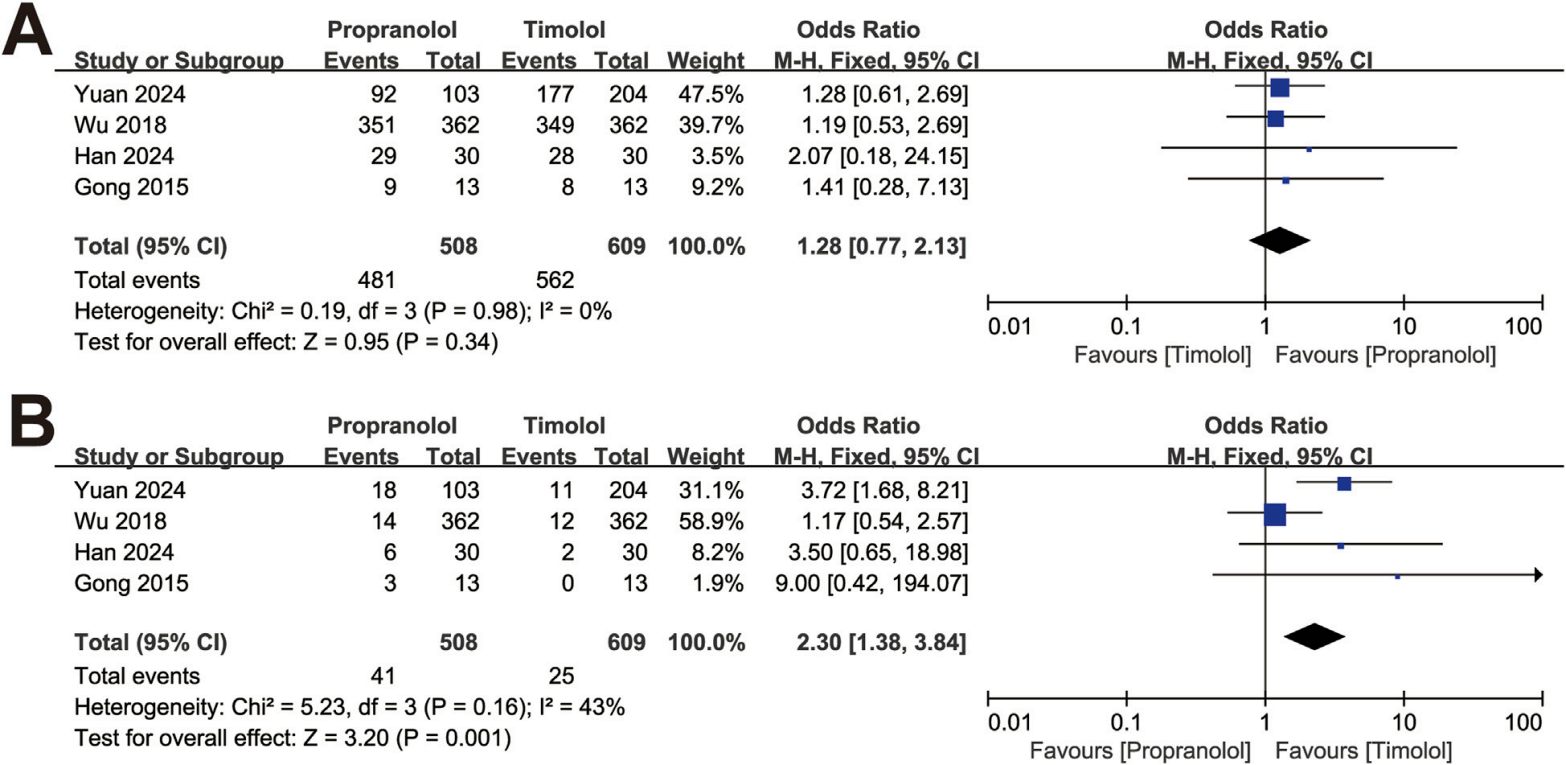
Forest plot of **(A)** efficacy in treating superficial IH and **(B)** adverse events of superficial IH.

The studies were divided into two subgroups by clinical classification: superficial and non-superficial IH. The analysis was conducted with the fixed-effect model. In four studies with superficial IH, including 508 patients treated with oral propranolol and 609 patients treated with topical timolol, the efficacy of the two treatments was comparable (OR = 1.28, 95% CI: 0.77–2.13; *P* = 0.34), with no significant heterogeneity (*P* for Cochrane’s Q test = 0.98, *I* ^*2*^ = 0%; Figure 3A). The pooled results revealed a significant difference in adverse events between the two treatment groups (OR = 2.30, 95% CI: 1.38–3.84; *P* = 0.001; Figure 3B).

**FIGURE 4:**
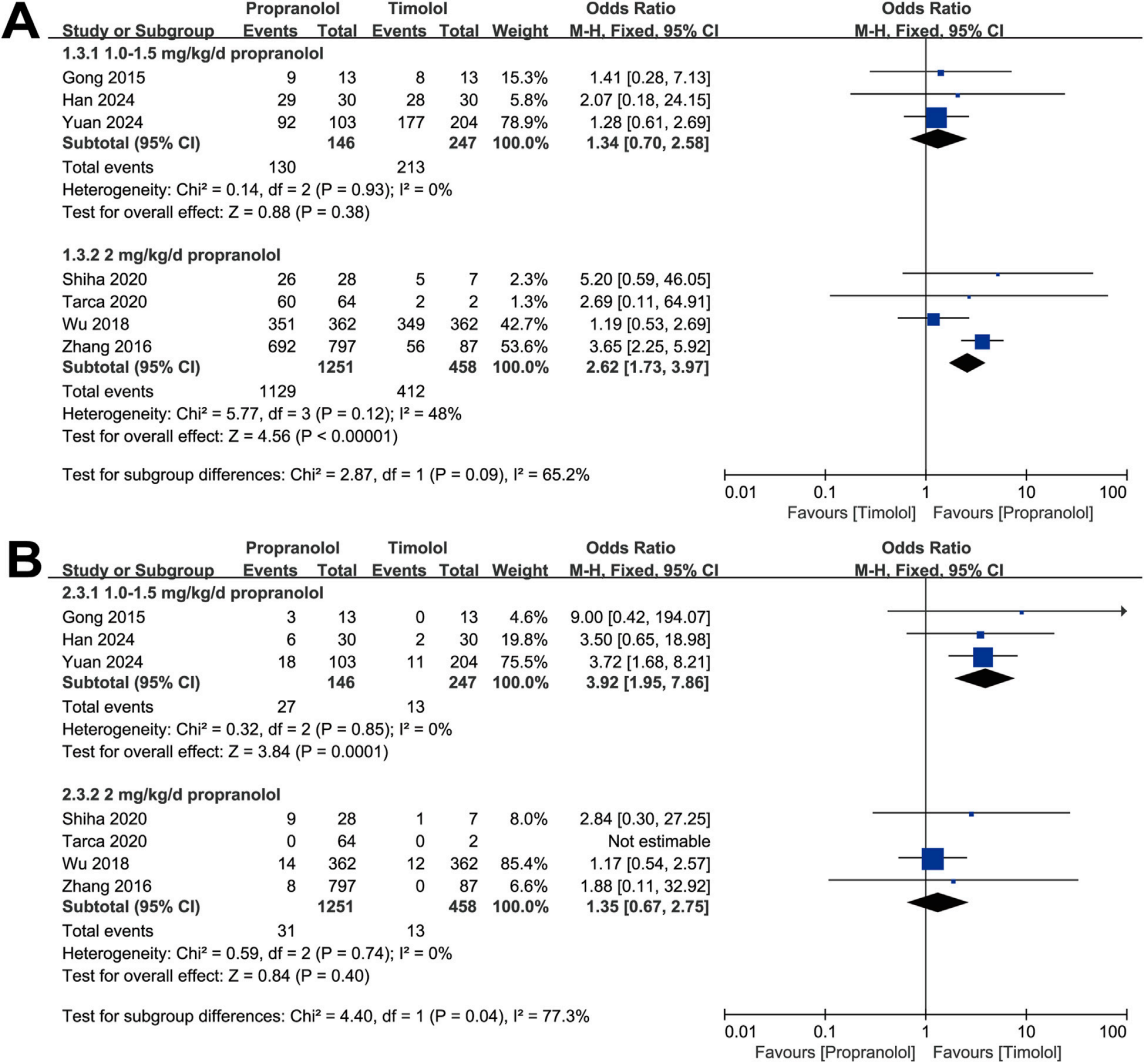
Forest plot of **(A)** efficacy and **(B)** adverse events associated with different dosages of propranolol.

Furthermore, we divided the studies according to the propranolol dosage into two subgroups: 1.0∼1.5 mg/kg/d and 2 mg/kg/d. The analysis was also conducted via a fixed-effect model. The results showed no significant difference in the efficacy of the 1.0∼1.5 mg/kg/d propranolol dosage group compared with the timolol group (OR = 1.34, 95% CI: 0.70–2.58; *P* = 0.38), with no significant heterogeneity (*P* for Cochrane’s Q test = 0.93, *I* ^*2*^ = 0%; Figure 4A). However, patients treated with topical timolol experienced fewer adverse events, indicating a greater safety profile (OR = 3.92, 95% CI: 1.95–7.86; *P* = 0.001; Figure 4B). Compared with timolol, treatment with 2 mg/kg/d propranolol resulted in a greater effective rate (OR = 2.62, 95% CI: 1.73–3.97; *P* < 0.001), with no significant heterogeneity (*P* for Cochrane’s Q test = 0.12, *I* ^*2*^ = 48%; Figure 4A), whereas adverse events between the two groups were comparable (OR = 1.35, 95% CI: 0.67–2.75; *P* = 0.40; Figure 4B).

### 3.6 Publication bias

**FIGURE 5:**
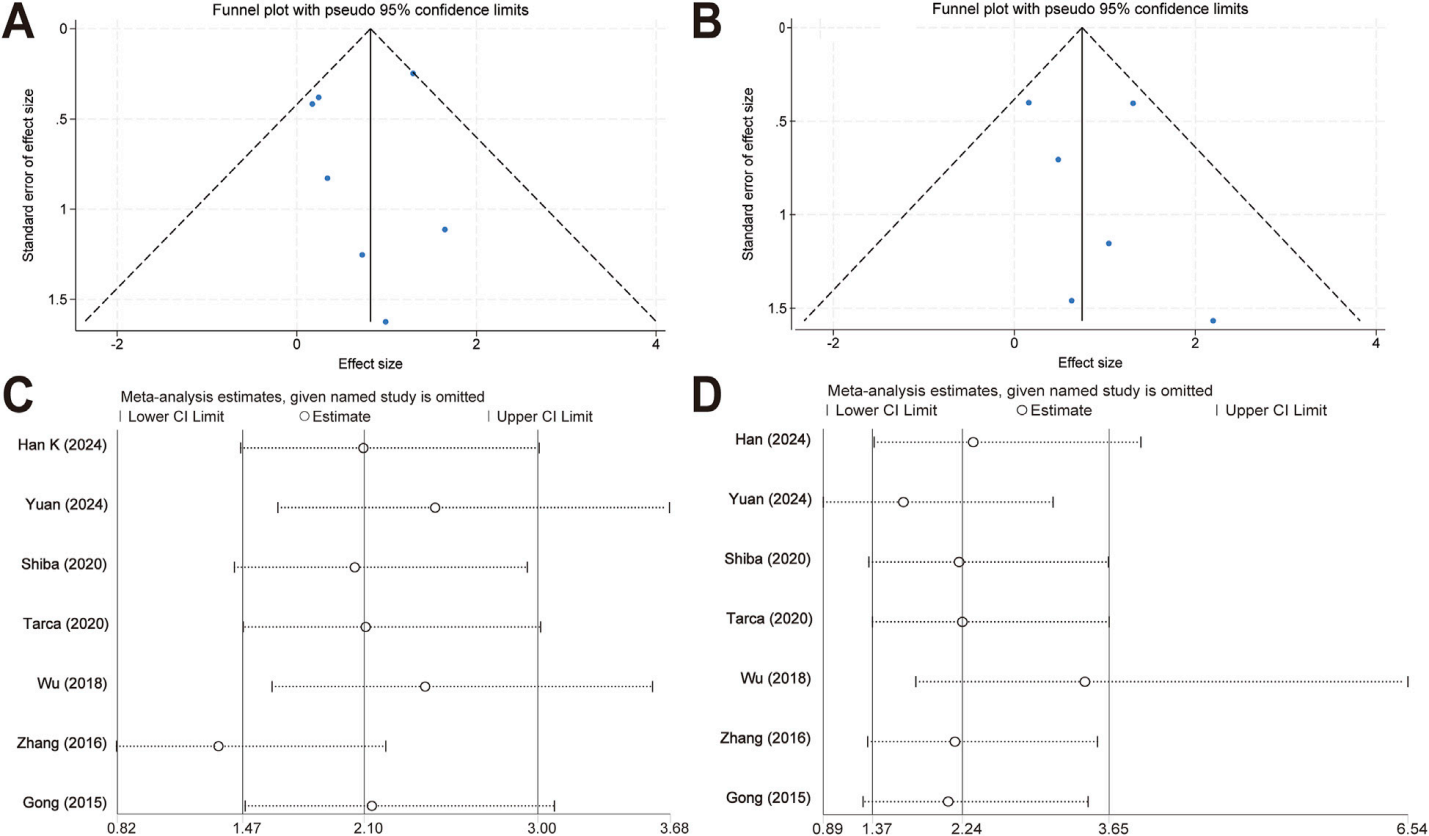
Publication bias evaluation and sensitivity analysis. **(A)** Funnel plots of response rates for the meta-analysis comparing topical timolol and oral propranolol for treating IH. **(B)** Funnel plots of safety outcomes for the meta-analysis comparing topical timolol and oral propranolol for treating IH. **(C)** Sensitivity analysis of response rates for the meta-analysis comparing topical timolol and oral propranolol for treating IH. **(D)** Sensitivity analysis of safety outcomes for the meta-analysis comparing topical timolol and oral propranolol for treating IH.

Funnel plots were created to estimate the publication bias of the included articles. As shown in Figures 5A, B, we found that the funnel plots of efficacy and safety between the two treatment groups were visually symmetric, suggesting that there was a low risk of publication bias.

### 3.7 Sensitivity analysis

The sensitivity analyses were performed by omitting one study at a time (Figures 5C, D). The ORs of the combined effect sizes did not change significantly when either study was excluded, suggesting that the results of the meta-analyses were relatively robust.

## 4 Discussion

Recently, a meta-analysis of RCTs revealed that, compared with oral propranolol, topical timolol has no difference in efficacy for treating IH but has a lower incidence of adverse events (Qiao et al., 2020). However, only 3 of the 8 included RCTs in this study were indexed in PubMed, Web of Science, the Cochrane Library, and Embase, which limits their retrievability. This limitation may affect the generalizability and reliability of the results and raise concerns about publication bias. Building on these limitations, our study included more studies than previously reported, aiming to provide more globally representative and methodologically consistent findings. Moreover, we conducted subgroup analyses to evaluate the safety and efficacy of propranolol versus timolol based on clinical classification (superficial vs. non-superficial) and propranolol dosage (1.0∼1.5 mg/kg/d vs. 2 mg/kg/d). Consistent with the previous meta-analysis and existing clinical outcomes, we found that the efficacy of oral propranolol was better than that of topical timolol, while propranolol resulted in a greater incidence of adverse events (Figure 2A). However, topical timolol can serve as an alternative to oral propranolol for treating superficial IH, providing similar efficacy with fewer adverse effects. In addition, our study confirmed that propranolol at a daily dose of 2 mg/kg has a better response rate than 1.0∼1.5 mg/kg/d, which aligns with existing clinical guidelines that recommend a dosage of 2∼3 mg/kg/d (Krowchuk et al., 2019; Mimura et al., 2020; Hoeger et al., 2015; Smithson et al., 2017). These findings indicate that for superficial IHs, it is recommended to use topical timolol for IH treatment. For deep or mixed IHs, although propranolol has more adverse events compared to timolol, studies have shown that adverse events with propranolol are well tolerated, and severe adverse events are rare (Leaute-Labreze et al., 2016; Prey et al., 2016; Droitcourt et al., 2018). Therefore, we recommend propranolol as the preferred treatment option.

For many years, systemic corticosteroids have been used as the mainstay for IH treatment (Satterfield and Chambers, 2019). Since propranolol was serendipitously observed to be effective in the treatment of severe IH in 2008, the role and mechanism of this drug have received extensive attention (Leaute-Labreze et al., 2017). Although the precise mechanism is unclear, propranolol is reported to promote IH regression by inducing vasoconstriction, inhibiting angiogenesis, and triggering apoptosis, all of which contribute to the reduction in the blood supply to the hemangioma and eventual shrinkage of the lesion (Rotter and de Oliveira, 2017). Specifically, by targeting mast cell β-adrenergic receptors, propranolol can promote HemECs autophagy and reduce blood VEGFA levels (Ye et al., 2022; Makkeyah et al., 2022). Moreover, R-propranolol isomers downregulate VEGF and angiopoietin-like 4 (ANGPTL4) in hemangioma stem cells (HemSCs), thus inhibiting tumor growth (Sasaki et al., 2019). Additionally, our previous study demonstrated that propranolol can suppress glycolysis in HemECs, thereby reducing the energy supply for IH growth (Yang et al., 2023). Clinically, propranolol has demonstrated superior efficacy in the treatment of IH, with high response rates and significant reductions in lesion size observed across numerous studies (Nagata et al., 2022; Pope et al., 2022; Leaute-Labreze et al., 2015).

Although oral propranolol is considered the first-line clinical treatment for IH (Tan et al., 2021), concerns about negative outcomes, including systemic adverse events, drug resistance, and relapses after withdrawal, have persisted over time (Han et al., 2024; Chang et al., 2016; Ahogo et al., 2013). Sleep disturbances, diarrhea, and bronchospasm induced by propranolol were reported in our included studies (Gong et al., 2015; Zhang et al., 2016; Sinha and Lloyd, 2020). Recently, Frongia et al. reported an 18% recurrence rate after oral propranolol treatment in their single-center retrospective study, which was particularly high in the head and neck area (Frongia et al., 2021). The limitations of propranolol in treating IH have prompted the search for safer alternatives, leading to the development and investigation of topical therapy. In this context, timolol has emerged as a promising option for reducing the risk of adverse reactions associated with systemic adverse events.

In 2010, a patient with periocular IH was successfully treated with 0.5% timolol maleate eye drops (Guo and Ni, 2010). Since then, numerous studies have demonstrated the efficacy and safety of topical timolol in treating IH, especially superficial IH (Lin et al., 2020; Xue and Hildebrand, 2013; Calvo et al., 2013; Rizvi et al., 2015). As a nonselective β-blocker, timolol was found to disrupt adrenergic signaling in the cornea, significantly inhibiting neovascularization and lymphangiogenesis of the cornea via the VEGF signaling pathway (Cho et al., 2018). However, research on the mechanisms of timolol in the treatment of IH is still limited, although Zhu et al. reported that timolol may increase the apoptosis rate of HemSCs (Zhu et al., 2023). Compared with propranolol, topical timolol has similar aesthetic outcomes and is well tolerated for IH treatment in clinical trials (Colmenero-Sendra et al., 2024; Munoz-Garza et al., 2021). Additionally, topical timolol is convenient with minimal stimulation, which enhances patient adherence. Furthermore, topical application avoids the potential issues associated with oral administration, such as bradycardia, hypoglycemia, and hypotension (Merino-Bohorquez et al., 2015).

However, several patients treated with topical timolol exhibit hyperkalemia, which may be related to the decrease in sodium‒potassium adenosine triphosphatase function caused by β2-receptor blockage (Alasmari et al., 2023; Al-Rwebah et al., 2020). Currently, topical timolol gel has been developed and put into clinical practice, exhibiting higher penetration rates than 0.5% timolol maleate eye drop does (Wu et al., 2017). Topical timolol gel is more stable with fewer adverse events and has been shown to achieve complete tumor regression (Merino-Bohorquez et al., 2015; Semkova and Kazandjieva, 2014). Furthermore, combination therapy with propranolol and timolol has been used in clinical practice and has demonstrated satisfactory efficacy with a reduced risk of side effects (Li et al., 2016; Kardasevic and Dinarevic, 2021; Mannschreck et al., 2019). However, owing to the absence of large RCTs, we did not evaluate the efficacy and safety of the combined treatment in our meta-analysis.

This study has several limitations. First, the included studies comprised RCTs, non-RCTs, and observational studies, which may have introduced confounding factors. RCTs minimize confounding variables through randomization, typically presenting a low risk of bias. However, non-RCTs and observational studies lack randomization and are therefore more susceptible to confounding. For example, baseline patient characteristics, such as age, comorbidities, and disease severity, may influence treatment allocation. To address these potential sources of bias, we conducted a literature quality assessment and a sensitivity analysis to ensure the robustness and generalizability of our results. The quality assessment showed that all studies had a low risk of bias and good overall methodological quality. Additionally, our sensitivity analysis, using a leave-one-out approach, confirmed that excluding any single study did not affect the combined ORs. These findings indicate robust results despite the inclusion of different study types. Second, the treatment strategies for IH varied, and the follow-up periods were relatively short. Four out of seven studies had follow-up periods of 1 year or less, limiting the ability to assess long-term treatment effects. While tumor regression may be evident in the short term, O'Brien et al. demonstrated that IHs can exhibit late recurrences after treatment cessation, even as late as 11 years of age (O’Brien et al., 2019). Therefore, large RCTs and long-term observations are needed in the future to fully assess the durability of treatment outcomes and identify any delayed effects or potential relapses after treatment cessation. Moreover, due to the limited number of eligible studies included in our meta-analysis (seven studies), quantitative methods such as Egger’s test or Begg’s test could not be performed to detect asymmetry in funnel plots. These methods require a sufficient number of studies to yield meaningful results, and when fewer than 10 studies are included in a meta-analysis, their statistical power is typically low, making them unreliable for detecting publication bias (Sterne et al., 2011). Therefore, although publication bias was visually assessed using funnel plots in our study, we acknowledge that the possibility of bias cannot be fully excluded due to the limited number of studies.

Additional limitations include the variable and relatively subjective evaluation of treatment response. For example, in the studies by Sinha et al. and Tarca et al., physicians assessed the treatment efficacy of propranolol solely by comparing the pretreatment and posttreatment photographs (Sinha and Lloyd, 2020; Tarca, 2020). Considering that propranolol is primarily used to treat deep or mixed IHs, it is therefore difficult to accurately evaluate its efficacy based solely on clinical observation, especially without additional metrics such as tumor volume or texture (Chang et al., 2017). Studies have also highlighted the importance of treatment duration in influencing the remission and recurrence rates of IH. Yang et al. reported that treatment durations exceeding 6 months result in a better response rate compared to shorter durations (Yang et al., 2019). For deep and combined IHs, it is recommended that optimal propranolol therapy extend through the entire proliferative phase of IHs and continue until at least 12 months of age to minimize relapses (Talaat et al., 2012). Furthermore, continued treatment for three additional months after achieving maximal regression significantly reduces His recurrence risk without increasing the rate of adverse events (Wang et al., 2024). Additionally, Holmes et al. reported a 24% rebound growth rate when propranolol treatment was stopped at an average age of 6.5 months (Holmes et al., 2011). These findings highlight the risk of premature treatment termination, which may result from subjective misjudgment and may adversely affect patient prognosis. To address these challenges, more objective outcome measures, including validated scoring systems (such as Achauer’s method, VAS method, and HAS method) and color Doppler ultrasound, have been introduced to enhance the reliability of treatment response assessments (Puttgen et al., 2016; Janmohamed et al., 2011; Achauer et al., 1997; Chang et al., 2017; Wang et al., 2024; Janmohamed et al., 2015). Additionally, several biomarkers, like VEGF, bFGF, and serum Apelin, have shown correlations with IH size during propranolol treatment, offering a promising avenue for more accurate and quantitative assessments (Park et al., 2020; Chen et al., 2024). These objective measures could play a crucial role in guiding decisions about continuing or discontinuing therapy with propranolol or timolol.

## 5 Conclusion

In conclusion, we systematically assessed the efficacy and safety of oral propranolol and topical timolol for the treatment of IH. Compared with topical timolol, oral propranolol demonstrated superior therapeutic efficacy in the treatment of IH. However, topical timolol can be considered an alternative for treating superficial IH, offering similar efficacy with fewer adverse effects. Additionally, 2 mg/kg/d propranolol has greater therapeutic efficacy with a comparable safety profile, while the 1.0∼1.5 mg/kg/d propranolol dosage shows similar efficacy to timolol but with a higher incidence of adverse events. In the future, more large-scale and multicenter RCTs are needed to validate and expand upon these findings.

## Data Availability

The original contributions presented in the study are included in the article/Supplementary Material, further inquiries can be directed to the corresponding authors.
