# Supplementary material for: Efficacy and safety of oral propranolol and topical timolol in the treatment of infantile hemangioma: a meta-analysis and systematic review
Source: Front Pharmacol. 2024 Dec 2;15:1515901. doi: 10.3389/fphar.2024.1515901 (PMC11646719; doi:10.3389/fphar.2024.1515901)
Supplement: Supplementary file 1 [file Table1.DOCX]

Supplementary Material

# Supplementary Table 1. Quality assessment of randomized controlled clinical trials using the Cochrane risk of bias tool.

| Study | Risk levels | Random sequence generation (selection bias) | Allocation concealment (selection bias) | Blinding of participants and personnel (performance bias) | Blinding of outcome assessment (detection bias) | Incomplete outcome data (attrition bias) | Selective reporting (reporting bias) | Other bias |
| --- | --- | --- | --- | --- | --- | --- | --- | --- |
| Gong(2015) | Low | Low | Unclear | Unclear | Low | Low | Low | Low |

Note: The risk of bias for each domain can be categorized into three levels: "Low" (low risk of bias), "some concerns" (some concerns) and " high risk" (high risk of bias). If the risk of bias evaluation results of all areas are "Low", then the overall risk of bias is "Low"; if the risk of bias evaluation results of some areas are "some concerns", then the overall risk of bias is "Low". If the risk of bias evaluation result of some areas is "some concerns" and there is no area of "high risk", then the overall risk of bias is "some concerns"; as long as there is an area where the risk of bias evaluation result is "low", then the overall risk of bias is "low". As long as there is one area where the risk of bias evaluation result is "high risk", then the overall risk of bias is "high risk".
