# Supplementary material for: Efficacy and safety of oral propranolol and topical timolol in the treatment of infantile hemangioma: a meta-analysis and systematic review
Source: Front Pharmacol. 2024 Dec 2;15:1515901. doi: 10.3389/fphar.2024.1515901 (PMC11646719; doi:10.3389/fphar.2024.1515901)
Supplement: Supplementary file 2 [file Table2.DOCX]

Supplementary Material

# Supplementary Table 2. Quality assessment of non-randomised controlled clinical trials using the ROBINS-I tool

| Study | Risk levels | Bias due to confounding | Bias in selection of participants into the study | Bias in classification of interventions | Bias due to deviations from intended interventions | Bias due to missing data | Bias in measurement of the outcome | Bias in selection of the reported results |
| --- | --- | --- | --- | --- | --- | --- | --- | --- |
| Han (2023) | low | low | low | low | low | low | low | low |
| Wu (2018) | low | moderate | no information | low | low | low | low | low |

Note: There are 5 levels of judgement: low risk of bias, moderate risk of bias, Serious risk of bias, Critical risk of bias, no information.
