# Supplementary material for: Efficacy and safety of oral propranolol and topical timolol in the treatment of infantile hemangioma: a meta-analysis and systematic review
Source: Front Pharmacol. 2024 Dec 2;15:1515901. doi: 10.3389/fphar.2024.1515901 (PMC11646719; doi:10.3389/fphar.2024.1515901)
Supplement: Supplementary file 3 [file Table3.DOCX]

Supplementary Material

# Supplementary Table 3. Risk of bias of all included observational studies using the Newcastle-Ottawa quality assessment scale.

| Author | Year | Selection | | | | Comparability | Outcome | | | Total score | Risk level^#^ |
| --- | --- | --- | --- | --- | --- | --- | --- | --- | --- | --- | --- |
|  |  | Representativenes s of the Exposed Cohort | Selection of the Non-Exposed Cohort | Ascertainment of Exposure | Demonstration That Outcome of Interest Was Not Present at Start of Study |  | Assessment of Outcome | Was Follow-Up Long Enough for Outcomes to Occur | Adequacy of Follow Up of Cohorts |  |  |
| Sinha | 2020 | 1 | 1 | 1 | 1 | 1 | 1 | 1 | 1 | 8 | low |
| Tarca | 2020 | 1 | 1 | 1 | 1 | 1 | 0 | 1 | 1 | 7 | low |
| Zhang | 2016 | 1 | 1 | 1 | 1 | 0 | 1 | 1 | 1 | 7 | low |
| Yuan | 2024 | 1 | 1 | 1 | 1 | 1 | 1 | 1 | 1 | 8 | low |

Note: ^#^Low (total score ≥ 7), moderate (total score 5-6), and high (total score ≤ 4) risk of bias.
